
JOURNAL INFORMATION
==============================
NLM Title Abbreviation: ScientificWorldJournal
Journal ID: ScientificWorldJournal
NLM Journal ID: 101131163
Journal ID: TSWJ

The Scientific World Journal

ISSN: 2356-6140
EISSN: 1537-744X
Publisher: Wiley

ARTICLE INFORMATION
==============================
PMCID: PMC5763698
PMID: 20563529
DOI: 10.1100/tsw.2010.102
Article ID: 360298
Article version: 1
Subjects: Directions in Science

Significance of the Syndecan-4-Transglutaminase-2 Interaction

Verderio Elisabetta *
Scarpellini Alessandra
School of Science and Technology, Nottingham Trent University, Nottingham, UK
*Elisabetta Verderio: elisabetta.verderio-edwards@ntu.ac.ukAcademic Editor: George Yip

Publication date: 2010
Electronic publication date: 2010 Jun 14
Volume: 10
First page: 1073
Last page: 1077
Received 2010 Mar 12; Revised 2010 Apr 29; Accepted 2010 May 5
Copyright: Copyright © 2010 Elisabetta Verderio and Alessandra Scarpellini.
Copyright year: 2010
License: This is an open access article distributed under the Creative Commons Attribution License, which permits unrestricted use, distribution, and reproduction in any medium, provided the original work is properly cited.
License URL: https://creativecommons.org/licenses/by/3.0/

Keywords: transglutaminase-2, syndecan-4, heparan sulphate proteoglycans, extracellular matrix, wound healing, fibrosis

Funding: Wellcome Trust 087163

==============================
Directions in Science
*TheScientificWorldJOURNAL* (2010) 10, 1073–1077
ISSN 1537-744X; DOI 10.1100/tsw.2010.102

TheScientificWorldJOURNAL
www.thescientificworld.com

### Significance of the Syndecan-4-Transglutaminase-2 Interaction

Elisabetta Verderio* and Alessandra Scarpellini
*School of Science and Technology, Nottingham Trent University, Nottingham, U.K.*

**E-mail** elisabetta.verderio-edwards@ntu.ac.uk

*Received March 12, 2010; Revised April 29, 2010; Accepted May 5, 2010; Published June 14, 2010*

---

**KEYWORDS**: transglutaminase-2, syndecan-4, heparan sulphate proteoglycans, extracellular matrix, wound healing, fibrosis

---

Transglutaminase type 2 (TG2) (EC 2.3.2.13) is a multifunctional, ubiquitous protein that has been implicated in the pathogenesis of many apparently unrelated diseases, such as tissue fibrosis[1,2], celiac disease[3], cancers[4,5], neurodegenerative disorders[6], and type II diabetes[7]. Although TG2 is capable of different biological activities, overall its expression and activation is believed to occur as a response to tissue injury and/or cell stress. Protein cross-linking is the enzymatic reaction for which TG2 is better known and depends on $Ca^{2+}$ binding and GTP dissociation from TG2, conditions which are favoured in the extracellular environment or following cell injury and loss of $Ca^{2+}$ homeostasis. Protein cross-linking leads to the formation of intra- or intermolecular Nε(γ-glutamyl)lysine bonds between the γ-carboxamide group of a peptide-bound Gln residue and either the ε-amino group of a peptide-bound Lys residue or a primary amino group of polyamines, resulting into protein polymerisation/modification[1]. TG2 causes additional post-translational modifications among which protein deamidation of Gln residues contributes to the development of disorders caused by gluten sensitivity (e.g., celiac disease)[3].

TG2-mediated protein cross-linking has been convincingly linked to normal and abnormal wound repair processes. General consensus exists that the externalisation of TG2 and activation of its cross-linking activity are responsible for extracellular matrix (ECM) stabilisation and resistance to matrix metalloproteinase degradation[8], and that uncontrolled cross-linking as a consequence of chronic cell insult and increased secretion of TG2 is implicated in the pathogenesis of tissue fibrosis[1,2]. Not all the externalised TG2 is believed to be enzymatically active, despite the favourable conditions of high $Ca^{2+}$/GTP in the extracellular environment[9]. Most TG2 is thought to remain latent and be activated on demand, e.g., for cell repair/proliferation in response to tissue injury/stress[2,10,11] or for modulation of the ECM[12,13,14], e.g., by transforming growth factor beta[15] in response to oxidative stress[16,17] and by the hypoxia inducible factor-1 in solid tumours[18]. TG2 cross-linking may be controlled by the tight binding of TG2 to large substrates like fibronectin (FN)[19], which can further complex with collagen, and by TG2 internalisation and subsequent lysosomal degradation[20]. In recent years, it has become clear that the influence of TG2 on cell-matrix interactions also depends on cross-linking independent mechanisms. TG2 has been ascribed an adhesive role by acting as either an intracellular GTPase[21], an integrin-$\beta_1$ co-receptor[22], or a structural protein supporting adhesion-mediated cell signalling independently from the Arg-Gly-Asp (RGD) cell binding domain of FN[23]. Despite the importance of TG2 externalisation for most of the cross-linking dependent and independent roles, the mechanism of TG2 secretion is elusive. TG2 lacks a leader peptide and is not post-translationally

*Corresponding author.
©2010 with author.
Published by TheScientificWorld; www.thescientificworld.com modified for classic externalisation through the endoplasmic reticulum/Golgi route[1]. Among the influencing factors are the requirements for TG2-active-state conformation[24,25] and an intact N-terminal FN binding site[26].

Recent studies by Scarpellini et al.[27] and Telci et al.[28] published in the *Journal of Biological Chemistry* have highlighted a novel interaction of TG2 with the heparan sulphate proteoglycan (HSPG) syndecan-4 (S4), and suggested that this interaction may be important for controlling the cell-surface trafficking and cross-linking activity of TG2[27], and for mediating the adhesion-dependent signalling role of matrix TG2[28].

Early work from Bergamini's group described affinity purification by heparin-sepharose as an effective step for TG2 purification from erythrocytes[29]. More recently, the involvement of cell-surface HSPGs in TG2-mediated RGD-independent cell adhesion was hypothesised[23], since pretreatment of cells with HS-degrading enzyme led to the disruption of this distinct cell adhesion process. This earlier work has prompted further investigations on the binding interaction between TG2 and heparin, a highly sulphated analogue of HS glycosaminoglycan chains, which only exist covalently bound to the core protein of cell-surface proteoglycans, e.g., syndecans, glypicans, and secreted proteoglycans[30]. HSPGs bind extracellular ligands through the HS and influence their biological activity by affecting protein stability, activity, conformation, as well as cell-surface localisation, membrane secretion/internalisation, and protein interactions[30,31]. Among the HSPG-subfamilies, the syndecans act as coreceptors for both ECM components and soluble ligands[32], and S4 has overlapping roles with extracellular TG2 in wound healing and fibrosis[33], thus suggesting the implication of syndecans in the biological function of TG2.

The hypothesis that S4/HS could regulate TG2 was recently tested using a range of *in vitro* and cell-based approaches[27]. Initially, TG2-heparin interaction was studied at equilibrium by heparin/HS solid-phase assays and in real time by surface plasmon resonance (SPR). The TG2-heparin/HS interaction was saturable and the apparent dissociation constant was in the low nM range ($K_D$ ~20 nM by solid phase and $K_D$ ~90 mM by SPR), suggesting high affinity of TG2 for heparin/HS, comparable to that for the classic binding partner FN[34]. Next, in cell systems, it was established that the coassociation of cell membrane TG2 with S4 is not mediated by FN (which binds both TG2 and HS), but largely depends on TG2 association with the HS chains of S4[27]. The significance of the TG2-S4 interaction was then studied in primary dermal fibroblasts using a combination of S4 knock-out/knock-in experiments. It was shown that lack of S4/HS leads to a lower level of cell-surface TG2 antigen and cross-linking activity, and a parallel accumulation of cytosolic TG2 in primary fibroblasts, but no changes in the total level of TG2 expression. Since heparin did not have a direct regulatory effect on TG2 enzymatic activity[27], this finding led to the suggestion that the HS chains of S4 may direct the cell-surface trafficking and localization of TG2 at cell-matrix adhesions. Indeed, complexes of TG2 and S4 were detected at cell-matrix adhesions by immunofluorescence[27]. Hence, cell-surface HS may affect the externalization of TG2 in a similar way to that recently reported for fibroblast growth factor-2[35]. Functional inhibition of HS with surfen, a small antagonist of HS[36], or heparitinase digestion led to even higher alterations in cell-surface TG2 activity than S4 knock-out, suggesting that more cell-surface HSPGs are likely to be implicated[27]. Since the endocytic receptor LRP1 responsible for TG2 internalisation has been shown to act in concert with HSPGs[20,31], the possibility of a dual function of HSPGs in TG2 externalisation and internalisation is intriguing. However, published data so far support a role for HSPGs in the trafficking of TG2 to the cell surface, since S4-null fibroblasts display an accumulation of intracellular TG2[27]. Given the variety of roles of HS, the high-affinity binding of TG2 for HS is likely to influence the function of TG2 widely in the extracellular environment. Consistent with this idea, Telci et al. recently showed that once TG2 is externalised and bound to FN, S4 may act as a cell-surface receptor mediating the RGD-independent outside-in signalling role of matrix TG2 in partnership with integrin-$\beta_1$[28]. The interaction of matrix TG2 with S4 has been shown to mediate activation of protein kinase C$\alpha$ and its further interaction with integrin-$\beta_1$, thus leading to activation of focal adhesion kinase and survival ERK1/2 mitogen activated protein kinases. This pathway, which is RGD-independent, relies on increased deposition of TG2 in the ECM, e.g., by cell secretion following cell lysis or erythrocyte rupture at wound sites. Hence, TG2-mediated RGD-independent cell adhesion is likely to be prevalent in restricted conditions of matrix breakdown, accumulation of RGD peptides of FN, and competitive block of integrin-mediated RGD-dependent pathway. It is envisaged that in these conditions, TG2 degradation would be limited by binding to both FN and HS[8,37,38]; hence, TG2 would be able to rescue cell adhesion and facilitate wound repair.

In conclusion, characterisation of the high-affinity binding of TG2 to HS has opened the way to new hypotheses on how TG2 may be externalised and its function regulated in the ECM. Studies so far have led to the identification of S4 as a new component of the cell-surface trafficking of TG2 and a novel cell-surface receptor for matrix TG2 (Fig. 1). It is anticipated that the flexibility and length of the HS chains would allow for this dual interaction of S4 with TG2 to take place. TG2 and S4 are molecules that are both increased in conditions of tissue fibrosis *in vivo*[2,39,40]. If their interaction is confirmed in *in vivo* situations, alteration of S4-TG2 association could become an attractive target for the control of tissue fibrosis.

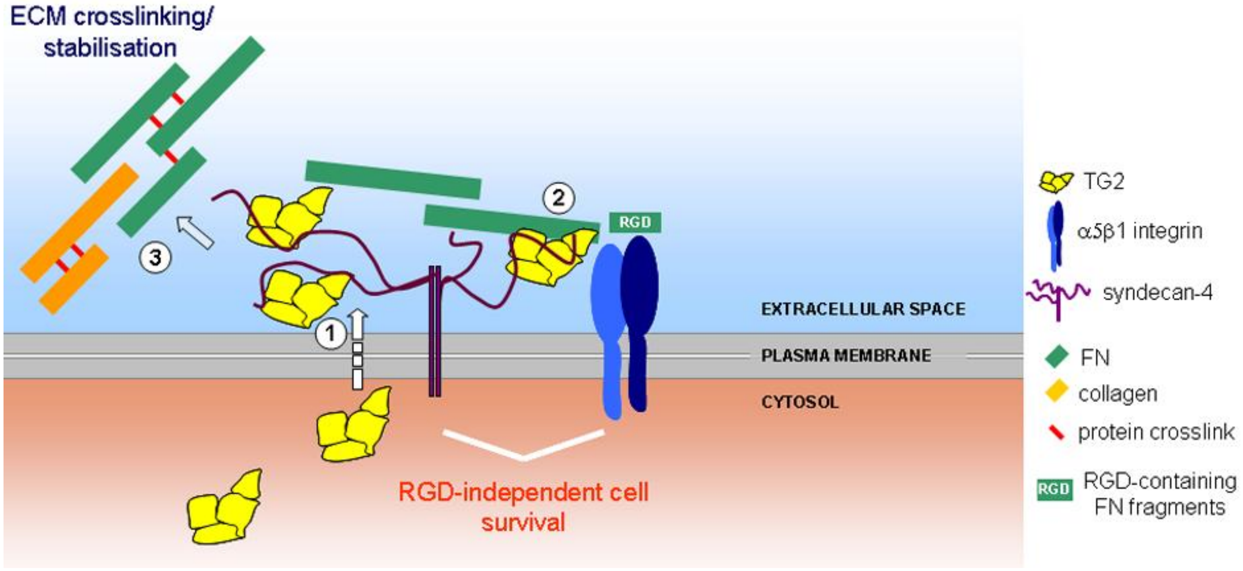

**FIGURE 1.** Double role of S4 in the cell-surface trafficking of TG2 and as a cell-surface receptor for matrix TG2. In primary dermal fibroblasts, the HS chains of S4 increase the concentration of cell-surface-associated TG2, suggesting their involvement in the cell-surface trafficking of TG2[27] (1). The mechanism of TG2 membrane targeting/translocation is unknown. Once externalised and deposited in the ECM, TG2 forms a tight complex with FN (2). In this form, TG2 is able to support RGD-independent cell-survival signalling by binding to the HS chains of S4, in partnership with integrin-β1, as a response to tissue damage/ECM degradation[28]. The externalised TG2 is also responsible for the modulation of the ECM through $Ca^{2+}$-dependent protein transamidation (3).

### ACKNOWLEDGEMENTS

E.V. is recipient of Wellcome Trust grant [087163]. We thank M. Griffin (Aston University, U.K.) and T. Johnson (University of Sheffield, U.K.) for critical comments.

### REFERENCES

1. Lorand, L. and Graham, R.M. (2003) Transglutaminases: crosslinking enzymes with pleiotropic functions. *Nat. Rev. Mol. Cell Biol.* **4**, 140–156.
2. Verderio, E.A., Johnson, T., and Griffin, M. (2004) Tissue transglutaminase in normal and abnormal wound healing: review article. *Amino Acids* **26**, 387–404.
3. Hadjivassiliou, M., Sanders, D.S., Grunewald, R.A., Woodroofe, N., Boscolo, S., and Aeschlimann, D. (2010) Gluten sensitivity: from gut to brain. *Lancet Neurol.* **9**, 318–330.

4. Mangala, L.S., Fok, J.Y., Zorrilla-Calancha, I.R., Verma, A., and Mehta, K. (2007) Tissue transglutaminase expression promotes cell attachment, invasion and survival in breast cancer cells. *Oncogene* **26**, 2459–2470.
5. Hwang, J.Y., Mangala, L.S., Fok, J.Y., Lin, Y.G., Merritt, W.M., Spannuth, W.A., Nick, A.M., Fiterman, D.J., Vivas-Mejia, P.E., Deavers, M.T., Coleman, R.L., Lopez-Berestein, G., Mehta, K., and Sood, A.K. (2008) Clinical and biological significance of tissue transglutaminase in ovarian carcinoma. *Cancer Res.* **68**, 5849–5858.
6. Jeitner, T.M., Pinto, J.T., Krasnikov, B.F., Horswill, M., and Cooper, A.J. (2009) Transglutaminases and neurodegeneration. *J. Neurochem.* **109(Suppl 1),** 160–166.
7. Bernassola, F., Federici, M., Corazzari, M., Terrinoni, A., Hribal, M.L., De Laurenzi, V., Ranalli, M., Massa, O., Sesti, G., McLean, W.H., Citro, G., Barbetti, F., and Melino, G. (2002) Role of transglutaminase 2 in glucose tolerance: knockout mice studies and a putative mutation in a MODY patient. *FASEB J.* **16**, 1371–1378.
8. Belkin, A.M., Akimov, S.S., Zaritskaya, L.S., Ratnikov, B.I., Deryugina, E.I., and Strongin, A.Y. (2001) Matrix-dependent proteolysis of surface transglutaminase by membrane-type metalloproteinase regulates cancer cell adhesion and locomotion. *J. Biol. Chem.* **276**, 18415–18422.
9. Siegel, M., Strnad, P., Watts, R.E., Choi, K., Jabri, B., Omary, M.B., and Khosla, C. (2008) Extracellular transglutaminase 2 is catalytically inactive, but is transiently activated upon tissue injury. *PLoS. One* **3**, e1861.
10. Haroon, Z.A., Hettasch, J.M., Lai, T.S., Dewhirst, M.W., and Greenberg, C.S. (1999) Tissue transglutaminase is expressed, active, and directly involved in rat dermal wound healing and angiogenesis. *FASEB J.* **13**, 1787–1795.
11. Nicholas, B., Smethurst, P., Verderio, E., Jones, R., and Griffin, M. (2003) Cross-linking of cellular proteins by tissue transglutaminase during necrotic cell death: a mechanism for maintaining tissue integrity. *Biochem. J.* **371**, 413–422.
12. Verderio, E., Nicholas, B., Gross, S., and Griffin, M. (1998) Regulated expression of tissue transglutaminase in Swiss 3T3 fibroblasts: effects on the processing of fibronectin, cell attachment, and cell death. *Exp. Cell Res.* **239**, 119–138.
13. Jones, R.A., Nicholas, B., Mian, S., Davies, P.J., and Griffin, M. (1997) Reduced expression of tissue transglutaminase in a human endothelial cell line leads to changes in cell spreading, cell adhesion and reduced polymerisation of fibronectin. *J. Cell Sci.* **110(Pt 19),** 2461–2472.
14. Gross, S.R., Balklava, Z., and Griffin, M. (2003) Importance of tissue transglutaminase in repair of extracellular matrices and cell death of dermal fibroblasts after exposure to a solarium ultraviolet A source. *J. Invest Dermatol.* **121**, 412–423.
15. Ritter, S.J. and Davies, P.J. (1998) Identification of a transforming growth factor-beta1/bone morphogenetic protein 4 (TGF-beta1/BMP4) response element within the mouse tissue transglutaminase gene promoter. *J. Biol. Chem.* **273**, 12798–12806.
16. Quan, G., Choi, J.Y., Lee, D.S., and Lee, S.C. (2005) TGF-beta1 up-regulates transglutaminase two and fibronectin in dermal fibroblasts: a possible mechanism for the stabilization of tissue inflammation. *Arch. Dermatol. Res.* **297**, 84–90.
17. Shin, D.M., Jeon, J.H., Kim, C.W., Cho, S.Y., Lee, H.J., Jang, G.Y., Jeong, E.M., Lee, D.S., Kang, J.H., Melino, G., Park, S.C., and Kim, I.G. (2008) TGFbeta mediates activation of transglutaminase 2 in response to oxidative stress that leads to protein aggregation. *FASEB J.* **22**, 2498–2507.
18. Jang, G.Y., Jeon, J.H., Cho, S.Y., Shin, D.M., Kim, C.W., Jeong, E.M., Bae, H.C., Kim, T.W., Lee, S.H., Choi, Y., Lee, D.S., Park, S.C., and Kim, I.G. (2010) Transglutaminase 2 suppresses apoptosis by modulating caspase 3 and NF-kappaB activity in hypoxic tumor cells. *Oncogene* **29**, 356–367.
19. Jeong, J.M., Murthy, S.N., Radek, J.T., and Lorand, L. (1995) The fibronectin-binding domain of transglutaminase. *J. Biol. Chem.* **270**, 5654–5658.
20. Zemskov, E.A., Mikhailenko, I., Strickland, D.K., and Belkin, A.M. (2007) Cell-surface transglutaminase undergoes internalization and lysosomal degradation: an essential role for LRP1. *J. Cell Sci.* **120**, 3188–3199.
21. Stephens, P., Grenard, P., Aeschlimann, P., Langley, M., Blain, E., Errington, R., Kipling, D., Thomas, D., and Aeschlimann, D. (2004) Crosslinking and G-protein functions of transglutaminase 2 contribute differentially to fibroblast wound healing responses. *J. Cell Sci.* **117**, 3389–3403.
22. Akimov, S.S., Krylov, D., Fleischman, L.F., and Belkin, A.M. (2000) Tissue transglutaminase is an integrin-binding adhesion coreceptor for fibronectin. *J. Cell Biol.* **148**, 825–838.
23. Verderio, E.A., Telci, D., Okoye, A., Melino, G., and Griffin, M. (2003) A novel RGD-independent cell adhesion pathway mediated by fibronectin-bound tissue transglutaminase rescues cells from anoikis. *J. Biol. Chem.* **278**, 42604–42614.
24. Balklava, Z., Verderio, E., Collighan, R., Gross, S., Adams, J., and Griffin, M. (2002) Analysis of tissue transglutaminase function in the migration of Swiss 3T3 fibroblasts: the active-state conformation of the enzyme does not affect cell motility but is important for its secretion. *J. Biol. Chem.* **277**, 16567–16575.
25. Telci, D., Collighan, R.J., Basaga, H., and Griffin, M. (2009) Increased TG2 expression can result in induction of TGF{beta}1 causing increased synthesis and deposition of matrix proteins which can be regulated by nitric oxide. *J. Biol. Chem.* **283**, 20937–20947.
26. Gaudry, C.A., Verderio, E., Aeschlimann, D., Cox, A., Smith, C., and Griffin, M. (1999) Cell surface localization of tissue transglutaminase is dependent on a fibronectin-binding site in its N-terminal beta-sandwich domain. *J. Biol. Chem.* **274**, 30707–30714.

27. Scarpellini, A., Germack, R., Lortat-Jacob, H., Muramatsu, T., Billett, E., Johnson, T., and Verderio, E.A. (2009) Heparan sulfate proteoglycans are receptors for the cell-surface trafficking and biological activity of transglutaminase-2. *J. Biol. Chem.* **284**, 18411–18423.
28. Telci, D., Wang, Z., Li, X., Verderio, E.A., Humphries, M.J., Baccarini, M., Basaga, H., and Griffin, M. (2008) Fibronectin-tissue transglutaminase matrix rescues RGD-impaired cell adhesion through syndecan-4 and beta1 integrin co-signaling. *J. Biol. Chem.* **283**, 20937–20947.
29. Signorini, M., Bortolotti, F., Poltronieri, L., and Bergamini, C.M. (1988) Human erythrocyte transglutaminase: purification and preliminary characterisation. *Biol. Chem. Hoppe Seyler* **369**, 275–281.
30. Bishop, J.R., Schuksz, M., and Esko, J.D. (2007) Heparan sulphate proteoglycans fine-tune mammalian physiology. *Nature* **446**, 1030–1037.
31. MacArthur, J.M., Bishop, J.R., Stanford, K.I., Wang, L., Bensadoun, A., Witztum, J.L., and Esko, J.D. (2007) Liver heparan sulfate proteoglycans mediate clearance of triglyceride-rich lipoproteins independently of LDL receptor family members. *J. Clin. Invest* **117**, 153–164.
32. Morgan, M.R., Humphries, M.J., and Bass, M.D. (2007) Synergistic control of cell adhesion by integrins and syndecans. *Nat. Rev. Mol. Cell Biol.* **8**, 957–969.
33. Verderio, E.A., Scarpellini, A., and Johnson, T.S. (2008) Novel interactions of TG2 with heparan sulfate proteoglycans: reflection on physiological implications. *Amino Acids* **36**, 671–677.
34. Hang, J., Zemskov, E.A., Lorand, L., and Belkin, A.M. (2005) Identification of a novel recognition sequence for fibronectin within the NH2-terminal beta-sandwich domain of tissue transglutaminase. *J. Biol. Chem.* **280**, 23675–23683.
35. Zehe, C., Engling, A., Wegehingel, S., Schafer, T., and Nickel, W. (2006) Cell-surface heparan sulfate proteoglycans are essential components of the unconventional export machinery of FGF-2. *Proc. Natl. Acad. Sci. U. S. A.* **103**, 15479–15484.
36. Schuksz, M., Fuster, M.M., Brown, J.R., Crawford, B.E., Ditto, D.P., Lawrence, R., Glass, C.A., Wang, L., Tor, Y., and Esko, J.D. (2008) Surfen, a small molecule antagonist of heparan sulfate. *Proc. Natl. Acad. Sci. U. S. A.* **105**, 13075–13080.
37. Lorand, L., Dailey, J.E. and Turner, P.M. (1988) Fibronectin as a carrier for the transglutaminase from human erythrocytes. *Proc. Natl. Acad. Sci. U. S. A.* **85**, 1057–1059.
38. Gambetti, S., Dondi, A., Cervellati, C., Squerzanti, M., Pansini, F.S., and Bergamini, C.M. (2005) Interaction with heparin protects tissue transglutaminase against inactivation by heating and by proteolysis. *Biochimie* **87**, 551–555.
39. Johnson, T.S., El-Koraie, A.F., Skill, N.J., Baddour, N.M., El Nahas, A.M., Njloma, M., Adam, A.G., and Griffin, M. (2003) Tissue transglutaminase and the progression of human renal scarring. *J. Am. Soc. Nephrol.* **14**, 2052–2062.
40. Yung, S., Woods, A., Chan, T.M., Davies, M., Williams, J.D., and Couchman, J.R. (2001) Syndecan-4 up-regulation in proliferative renal disease is related to microfilament organization. *FASEB J.* **15**, 1631–1633.

---

**This article should be cited as follows:**

Verderio, E. and Scarpellini, A. (2010) Significance of the syndecan-4-transglutaminase-2 interaction. *TheScientificWorldJOURNAL* **10**, 1073–1077. DOI 10.1100/tsw.2010.102.

---
